# Genetic relationships and evolution of old Chinese garden roses based on SSRs and chromosome diversity

**DOI:** 10.1038/s41598-017-15815-6

**Published:** 2017-11-13

**Authors:** Jiongrui Tan, Jing Wang, Le Luo, Chao Yu, Tingliang Xu, Yuying Wu, Tangren Cheng, Jia Wang, Huitang Pan, Qixiang Zhang

**Affiliations:** Beijing Key Laboratory of Ornamental Plants Germplasm Innovation & Molecular Breeding, National Engineering Research Center for Floriculture, Beijing Laboratory of Urban and Rural Ecological Environment, Key Laboratory of Genetics and Breeding in Forest Trees and Ornamental Plants of Ministry of Education and College of Landscape Architecture, Beijing Forestry University, Beijing, 100083 China

## Abstract

Old Chinese garden roses are the foundation of the modern rose, which is one of the best-selling ornamental plants. However, the horticultural grouping and evolution of old Chinese garden roses are unclear. Simple sequence repeat (SSR) markers were employed to survey genetic diversity in old Chinese garden roses and genetic differentiation was estimated among different rose groups. Fluorescence *in situ* hybridization was used to study the physical localization of 5 S rDNA genes and a karyotype analysis was performed. The SSR data suggest that old Chinese garden roses could be divided into Old Blush group, Odorata group and Ancient hybrid China group. The Old Blush group had the most primitive karyotype. The Ancient hybrid China group and modern rose had the most evolved karyotypes and the highest genetic diversity. During the evolution of rose cultivars, 5 S rDNA increased in number, partially weakened in signal intensity and exhibited variation in distance from the centromere. In conclusion, rose cultivars evolved from the Old Blush Group to the Odorata group, the Ancient Hybrid China group and the modern rose. This work provides a basis for the collection, identification, conservation and innovation of rose germplasm resources.

## Introduction

Plant breeding aims to combine traits of interest with existing traits. The conservation and innovation of germplasm resources are the foundation of breeding programs. There are more than 24000 rose cultivars, and these are among most popular ornamental plants^1^. The cultivation of roses has a long history and can be traced back to Roman antiquity and even 3000 BC in China^2,3^. China is the distribution centre of the genus *Rosa*. Since early 19th century, continuous-flowering, tea-scented and crimson old Chinese garden roses were successively introduced into Europe, triggering a new era of modern roses^4^. Old Chinese garden roses were of great importance in the background of modern roses owing to the specific traits they contributed^3,5^. Old Chinese garden roses were bred and cultivated since the Song Dynasty (960–1279 BC)^6^; they experienced a variety of natural disasters and wars, and survived for the past one thousand years. Old Chinese garden roses and wild species are the basis for a breeding approach to improve adaptability and disease resistance and to enrich the narrow gene pool of modern roses^7^.

Cultivated roses are mostly horticulturally classified into three groups based on phenotypic characters: (i) wild species or botanical roses, (ii) old garden roses that existed prior to 1867 and (iii) modern roses^8^. There are two subdivisions of old garden roses from China (China roses and Tea roses) and several subdivisions of old garden roses with a genetic background influenced by China roses, such as Bourbon, Noisette and Hybrid Perpetual^9^. The present classification is used as a reference, but requires continuous evaluation^9^. China roses, as a subdivision of old garden roses, refer to the group that includes *Rosa chinensis* in sect. Chinenses as well as its horticultural varieties, and early Hybrid China roses are characterized by a moderate fragrance, continuous-flowering, and have been introduced to western Europe since the 18th century^8,10^. Tea roses (i.e. Tea-scented China roses) are continuous-flowering roses, named for their scent, which resembles that of Chinese black tea; they have individual flowers with petals that tend to roll back at the edge^8^.

Both China roses and Tea roses are everblooming erect shrubs belonging to old Chinese garden roses, but they do not represent all of the old Chinese garden rose germplasm. There are many cultivars with diverse phenotypic characteristics within old Chinese garden roses, including once blooming and climbing characteristics. For rose germplasm innovation, it is crucial to clarify the grouping, genetic relationships, and early breeding process of old Chinese garden roses. However, the horticultural grouping and evolution of old Chinese garden roses are unclear. Li *et al*. reported that old Chinese garden roses could be clustered into six groups based on morphological characteristics^11^. The Old Blush group and *Rosa odorata* group have been identified when classifying old Chinese garden roses based on morphological characteristics^12^. Soules found eight synonyms or sports of ‘Old Blush’ among China roses based on identical simple sequence repeat (SSR) profiles and named them the Old Blush group^10^. In order to improve the grouping of old Chinese garden roses, additional cytogenetic and molecular analyses are needed.

Molecular marker technologies have been used to study the genetic relationships among groups of roses^5,8,10,13–16^. Cytogenetic analyses have also been used for taxonomic, evolution and speciation analyses of the genus *Rosa* ^17–21^. Despite rDNA-FlSH (fluorescence *in situ* hybridization) analyses of the genetic relationships among several cultivars and species in the subgenus *Rosa* ^22–30^, the physical positions of rDNA have rarely been used in studies of the evolutionary relationships among rose cultivars^31^. Molecular markers and FISH have been combined to analyse the genetic variability of rose cultivars and species^32^. However, few studies have combined cytogenetic techniques and molecular biology techniques to analyse the genetic relationships and evolution of old Chinese garden roses.

As an important germplasm resource of modern roses, further studies of old Chinese garden roses can not only improve our understanding of the genetic background of modern roses, but can also contribute to the identification, collection, preservation and innovation of rose germplasm resources. Therefore, we combined SSR markers and FISH to analyse the genetic relationships and evolution of old Chinese garden roses. For the purposes of this study, old Chinese garden roses can be divided into three groups: Old Blush group (varieties and cultivars of *R. chinensis* var. *chinensis*), Odorata group (varieties and cultivars of *R. odorata*) and Ancient Hybrid China group (cultivars of *R. chinensis*). The goal was to test the following hypotheses: (i) the Old Blush group is genetically distinct from other ever-blooming old Chinese garden roses; (ii) the Ancient hybrid China group is derived from the Old Blush group, Odorata group and species; and (iii) the physical locations of 5 S rDNA genes on chromosomes are related to the genetic relationships and karyotype evolution of rose cultivars.

## Results

### Microsatellite marker analysis

Twenty-two SSRs (see Supplementary Table S2) were used to identify 81 genotypes (see Supplementary Table S1); these SSRs were highly polymorphic, with 4 to 19 alleles per marker and a total of 227 alleles over 22 primer pairs. All markers used in the study have been mapped in the final integrated map for ‘Yunzheng Xiawei’ and ‘Sun City’ (LG2-LG7) and preliminary linkage groups of ‘Yunzheng Xiawei’ (Y4, Y12)^33^. *A*_*m*_ ranged from 1.3 alleles for 464 to 2.2 alleles for Rw22A3. *A*_*e*_ ranged from 0.9 for Rw22A3 to 1.9 for 397. Gene diversity (*H*_*e*_) ranged from 0.281 to 0.865. Generally, markers with fewer alleles had lower *H*_*e*_ values, except 637 and 327, which had 5 and 8 alleles, respectively, but *H*_*e*_ values of 0.735 and 0.798. An exception was marker 509, which had 13 alleles, but a relatively low *H*_*e*_ value (*H*_*e*_ = 0.442) and *A*_*e*_ value (*A*_*e*_ = 1.3). This indicates that marker 509 had a high proportion of low frequency alleles.

### Cluster analysis using molecular markers

**Figure 1:**
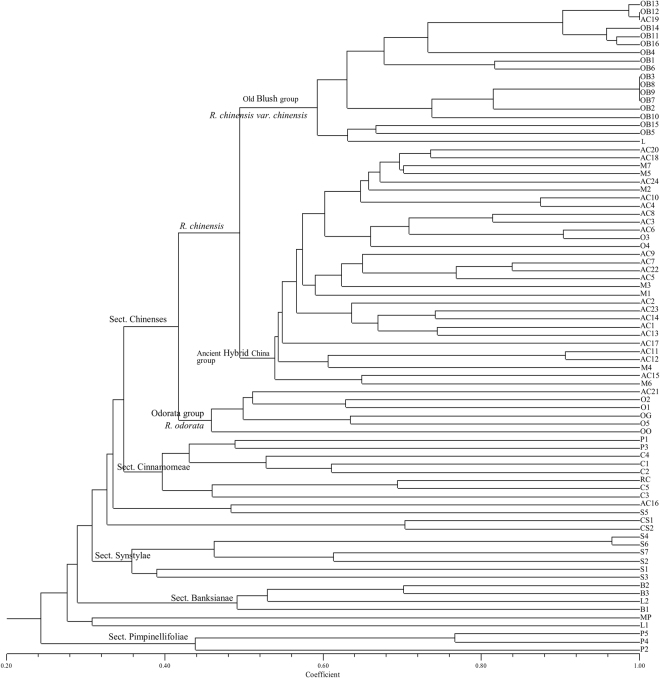
UPGMA dendrogram obtained from a cluster analysis of 81 rose accessions based on 22 SSRs. Note: This dendrogram was produced using the Unweighted Pair Group Method with Arithmetic Mean clustering from the Dice similarities of the SSR data. The main groups of interest are indicated near the top node of the cluster.

As shown in the dendrogram in Fig. 1, the cultivars and species of sect. Chinenses were separated from other sections of the subgenus *Rosa* with a similarity coefficient of approximately 0.34 and formed well-defined groups at a similarity of approximately 0.42. The dendrogram clusters generally conformed to the current classification. The genetic diversity for all accessions was high, with similarity coefficients for non-identical samples ranging from ~0.22–0.99.

The first group in the dendrogram is the Old Blush group. They are contained within the largest cluster, which also contains sub-clusters of the Ancient Hybrid China group and the Odorata group. Based on the similarity coefficients, the accessions in the Old Blush group are closely related, and the genetic diversity is lower for these accessions, which are cultivars of *R. chinensis* var. *chinensis*. *R. lucidissima* (L) clustered in the Old Blush group. *R. lucidissima* clustered with ‘Zhaiye Tengben Yuejihua’ and ‘Teng Yueyue Hong’ (OB15 and OB5) with a similarity coefficient of 0.63; they are all climbing shrubs. The top six accessions of the Old Blush group clustered together with a similarity coefficient of greater than 0.90. The genetic diversity of this group was very low. ‘Viridiflora’ (AC19) and ‘Yueyue Fen’ (OB12) had identical SSR profiles.

The largest cluster within this dendrogram separated into two groups at a similarity of approximately 0.42. The deeper part of the largest cluster generally included climbing shrubs classified as *R. odorata* varieties, including their wild ancestor *R. odorata* var. *gigantea* (OG). The genetic diversity of this cluster was lower than those of the Old Blush group and Ancient Hybrid China group, with similarity coefficients of ~0.46–0.64. ‘Siji Danhuang Xiangshui Yueji’ (O3) and ‘Siji Fenhong Xiangshui Yueji’ (O4) were not observed in the Odorata group; O3, namely ‘Parks’ Yellow Tea-Scented China’, and O4 are ever blooming Tea roses and have a high heterozygosity.

There was a cluster of cultivars between the Old Blush group and Odorata group, most of which were ancient hybrid China rose cultivars. The common characteristics of these Ancient Hybrid China roses are erect shrubs and recurrent blooming. According to the similarity coefficient between samples within groups, the Ancient Hybrid China group has greater levels of genetic diversity than those of other groups. The range of similarity coefficients for this cluster (about 0.54–0.90) was intermediate to those of the Old Blush group (about 0.60–0.99) and Odorata group (about 0.46–0.64). Seven modern roses belonged to this group as well, including Hybrid Tea and Floribunda. The cluster adjacent to sect. Chinenses contained all accessions of sect. Cinnamomeae and a cultivar of *R. rugosa*, ‘Dahong Zizhi’ (HR), which is the progeny of interspecific hybridization in sect. Cinnamomeae, *R. rugosa* × *R. davurica*. Sect. Synstylae taxa clustered together, except *R. multiflora* var*. carnea* (S5), which is separated by *R. chinensis* var. *spontanea* (CS1 and CS2). The last two clear clusters were sect. Banksianae and sect. Pimpinellifoliae.

### Genetic differentiation among groups

Genetic differentiation between the Old Blush group and Odorata group and between the Old Blush group and Ancient Hybrid China group was moderate (*F*_ST_ = 0.10, 0.11) (Table 1), indicating that the Old Blush group is distinct from other recurrent blooming Ancient Hybrid China roses and cultivars of the Odorata group. The Old Blush group and species in sect. Chinenses had high genetic differentiation (*F*_ST_ = 0.15) and the Old Blush group and sect. Synstylae, Old Blush group and sect. Cinnamomeae, and Old Blush group and other sections exhibited very high genetic differentiation (*F*_ST_ = 0.26, 0.34 and 0.31, respectively), indicating that the Old Blush group is genetically more closely related to species in sect. Chinenses than sect. Synstylae, sect. Cinnamomeae and other sections of the subgenus *Rosa*.

**Table 1:** Genetic differentiation (*F*_ST_) among rose types of 81 rose accessions based on 22 SSRs. OB, Old Blush group; O, Odorata group, AC, Ancient Hybrid China group; M, modern roses; SSC, Species roses in sect. Chinenses; S, sect. Synstylae; SC, sect. Cinnamomeae; OS, Other sections.

	OB	O	AC	M	SSC	S	C	OS
OB								
O	0.10							
AC	0.11	0.05						
M	0.20	0.08	0.01					
SSC	0.15	0.05	0.06	0.07				
S	0.26	0.15	0.10	0.07	0.04			
C	0.34	0.23	0.15	0.12	0.13	0.13		
OS	0.31	0.16	0.14	0.11	0.13	0.10	0.07	

The shading varies from white to dark grey according to the height of the *F*_ST_ value. A high *F*_ST_ means a high distance between groups. 0.0 < *F*_ST_ *< *0.05: little genetic differentiation; 0.05 < *F*_ST_ < 0.15: moderate genetic differentiation; 0.15 < *F*_ST_ < 0.25: high genetic differentiation; *F*_ST_ > 0.25: very high genetic differetiation^64^.

The *F*_ST_ values for the Ancient Hybrid China group and Old Blush group, Ancient Hybrid China group and Odorata group, Ancient Hybrid China group and species in sect. Chinenses, Ancient Hybrid China group and sect. Synstylae, and Ancient Hybrid China group and other sections all indicated moderate genetic differentiation (Table 1). Accordingly, the breeding process of the Ancient Hybrid China group may involve the Old Blush group, Odorata group, species in sect. Chinenses, sect. Synstylae and other sections of the subgenus *Rosa*. The genetic distance between the Ancient Hybrid China group and modern roses was the lowest (*F*_ST_ = 0.01) (Table 1), suggesting that they share a similar genetic background. These findings are consistent with the dendrogram, indicating that the Ancient Hybrid China group and modern roses are not two genetically independent groups. *F*_ST_ values for comparisons between species of roses not in sect. Chinenses (sect. Synstylae, sect. Cinnamomeae and Other sections) and each horticultural rose group were as follows: Old Blush group > Odorata group > Ancient Hybrid China group > modern roses (Table 1). The degree of heterozygosity increased from the Old Blush group to Odorata group, Ancient Hybrid China group and modern roses, and this order may reflect the evolution of these groups, to some extent.

### Karyotype analysis

The metaphase chromosome karyotypes were obtained by fluorescence *in situ* hybridization. Ploidy levels ranged from 2x to 4x, with a basic chromosome number of x = 7 (Table 2). No aneuploidy was observed in the tested materials. The chromosomes are compiled in Table 2. Cultivars of the Old Blush Group and *R. odorata* were all diploid, and some members of the Ancient Hybrid China group were also diploid. Triploids and tetraploids exist in the Ancient Hybrid China group and Modern Roses as a result of artificial domestication and distant hybridization. The polyploidy in the Ancient Hybrid China group and modern roses indicates a higher level of evolution than that of the Old Blush Group and *R. odorata*.

**Table 2:** Karyotype parameters for 19 rose cultivars.

Sample number	Cultivar or species	Arm ratio	Lt/St	Relative length of chromosome	Formula of karyotype	Karyotype	Asymmetrical karyotypeindex
OB12	‘Yueyue Fen’	1.02 ± 0.05~2.30 ± 0.03	1.40 ± 0.005	2 n = 8 M1 + 6 M2	2 n = 2 x = 14 = 12 m + 2 sm	2 A	56.70 ± 0.13%
OB13	‘Yueyue Hong’	1.26 ± 0.02~1.90 ± 0.06	1.47 ± 0.009	2 n = 8 M1 + 6 M2	2 n = 2 x = 14 = 12 m + 2 sm	1 A	59.47 ± 0.16%
OB15	‘ZhaiyeTengbenYuejihua’	1.40 ± 0.01~2.51 ± 0.02	1.76 ± 0.012	2 n = 8 M1 + 4 M2 + 2 L	2 n = 2 x = 14 = 10 m + 4 sm	2 A	63.42 ± 0.11%
O2	‘Danhuang Xiangshui Yueji’	1.51 ± 0.03~2.10 ± 0.06	1.61 ± 0.016	2 n = 8 M1 + 4 M2 + 2 L	2 n = 2 x = 14 = 4 m + 10 sm	2 A	64.56 ± 0.12%
OO	*R. odorata var. odorata*	1.47 ± 0.03~2.45 ± 0.05	1.86 ± 0.018	2 n = 4 S + 2 M1 + 6 M2 + 2 L	2 n = 2 x = 14 = 4 m + 10 sm	2 A	65.95 ± 0.15%
AC1	‘Bao Xiang’	1.43 ± 0.04~2.39 ± 0.02	2.06 ± 0.017	2 n = 3 S + 6 M1 + 9 M2 + 3 L	2 n = 3 x = 21 = 15 m + 6 sm	2B	63.28 ± 0.15%
AC6	‘Huzhong Yue’	1.31 ± 0.01~1.84 ± 0.03	3.10 ± 0.010	2 n = 4 S + 4 M1 + 4 M2 + 2 L	2 n = 2 x = 14 = 12 m + 2 sm	1B	60.04 ± 0.18%
AC7	‘Jinfen Lian’	1.19 ± 0.03~1.84 ± 0.01	1.99 ± 0.012	2 n = 4 S + 8 M1 + 12 M2 + 4 L	2 n = 4 x = 28 = 24 m + 4 sm	1 A	60.24 ± 0.17%
AC11	‘Mutabilis’	1.35 ± 0.03~1.96 ± 0.03	2.01 ± 0.007	2 n = 2 S + 4 M1 + 6 M2 + 2 L	2 n = 2 x = 14 = 10 m + 4 sm	1B	61.89 ± 0.21%
AC16	‘Sai Zhaojun’	1.23 ± 0.01~2.00 ± 0.03	1.66 ± 0.013	2 n = 2 S + 6M1 + 6M2	2 n = 2 x = 14 = 8 m + 6 sm	1 A	61.35 ± 0.08%
AC18	‘Si Chun’	1.31 ± 0.05~2.46 ± 0.04	2.46 ± 0.020	2 n = 2 S + 6 M1 + 4 M2 + 2 L	2 n = 2 x = 14 = 6 m + 8 sm	2B	64.64 ± 0.19%
AC19	‘Viridiflora’	1.13 ± 0.04~2.13 ± 0.03	1.87 ± 0.013	2 n = 2 S + 6 M1 + 4 M2 + 2 L	2 n = 2 x = 14 = 10 m + 4 sm	2 A	62.16 ± 0.13%
AC20	‘Yingri Hehua’	1.39 ± 0.02~2.17 ± 0.02	2.30 ± 0.013	2 n = 3 S + 9 M1 + 6 M2 + 3 L	2 n = 3 x = 21 = 12 m + 9 sm	2B	63.11 ± 0.17%
AC21	‘Yu Linglong’	1.31 ± 0.05~1.96 ± 0.03	2.48 ± 0.020	2 n = 2 S + 6 M1 + 4 M2 + 2 L	2 n = 2 x = 14 = 8 m + 6 sm	1B	62.85 ± 0.12%
AC24	‘Zi Xiang Rong’	1.21 ± 0.03~2.26 ± 0.02	2.04 ± 0.015	2 n = 6 S + 6 M1 + 6 M2 + 3 L	2 n = 3 x = 21 = 9 m + 12 sm	2B	64.34 ± 0.13%
M1	‘Betty Prior’	1.48 ± 0.03~2.30 ± 0.04	1.90 ± 0.006	2 n = 6 S + 6 M1 + 3 M2 + 6 L	2 n = 3 x = 21 = 6 m + 15 sm	2 A	64.94 ± 0.14%
M3	‘Goldmarie’	1.45 ± 0.01~1.98 ± 0.03	3.01 ± 0.015	2 n = 4 S + 8 M1 + 12 M2 + 4 L	2 n = 4 x = 28 = 24 m + 4 sm	1B	62.20 ± 0.15%
M4	‘Honglian Wu’	1.33 ± 0.02~1.99 ± 0.04	2.12 ± 0.010	2 n = 3 S + 12 M1 + 3 M2 + 3 L	2 n = 3 x = 21 = 15 m + 6 sm	1B	60.88 ± 0.16%
M7	‘Princesse de Monaco’	1.48 ± 0.04~2.22 ± 0.05	2.27 ± 0.023	2 n = 4 S + 12 M1 + 8 M2 + 4 L	2 n = 4 x = 28 = 16 m + 12 sm	2B	64.24 ± 0.18%

Four karyotypes were observed in the tested rose materials: 1 A, 2 A, 1B and 2B (Table 2). Rose karyotypes of the Old Blush group and *R. odorata* were mainly 1 A and 2 A, while the karyotypes of Ancient Hybrid China roses and modern roses were mainly 1B and 2B. According to the classification standard of plant karyotype symmetry, karyotype asymmetry is high when the asymmetrical karyotype index is greater than 60%, and a karyotype is more primitive when the karyotype asymmetry coefficient is closer to 50%^34^. Only two asymmetrical karyotype indexes were less than 60%, i.e. ‘Yueyue Fen’ and ‘Yueyue Hong’ (Table 2), indicating that they are the most primitive roses among all tested materials.

**Figure 2:**
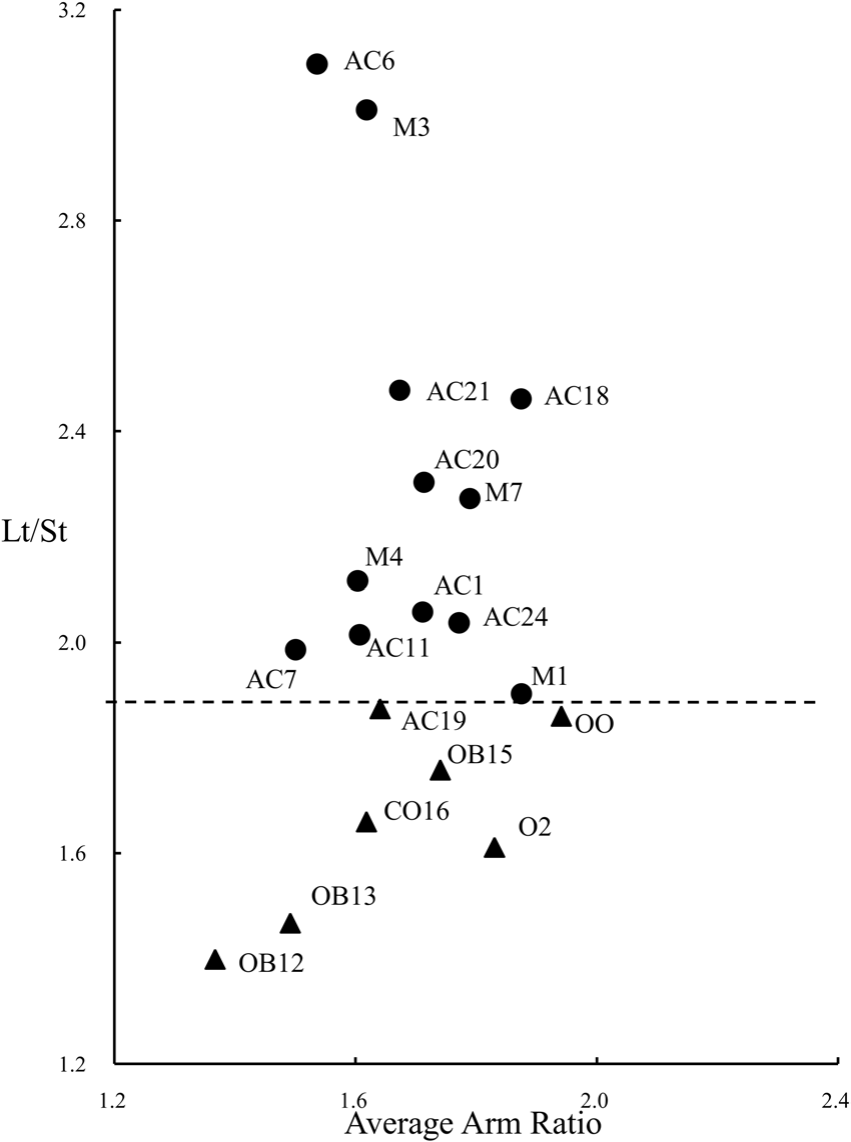
Scatter diagram of 19 rose cultivars based on the degree of karyotype asymmetry. The triangles represent diploid rose cultivars (Lt/St < 1.9).

Only the m and sm chromosome types were detected in the tested materials (Table 2). To visually compare the karyotype asymmetry between different roses, a scatter plot of the average arm ratio on the *X*-axis against the longest and the shortest chromosome ratio on the *Y*-axis was obtained (Fig. 2). The relative position of the coordinate points in the scatter plot reflect the asymmetry, the degree of evolution and the relationships among rose cultivars. For points close to the upper righthand corner, the karyotype is more asymmetrical, and the degree of cultivar evolution is high. In the opposite region, the degree of evolution of cultivars is lower. As shown in Fig. 2, ‘Yueyue Fen’ (OB12) in the lower left-hand corner is the most primitive cultivar.

Points in the lower part of the plot represent diploid cultivars (Lt/St < 1.9) with karyotype 1 A or 2 A (Fig. 2), indicating that they are more primitive with respect to the chromosome ratio. However, the evolution of these cultivars was essentially synchronous with respect to the chromosome ratio and the average arm ratio. ‘Viridiflora’ (AC19) and ‘Zhaiye Tengben Yuejihua’ (OB15) were more highly evolved than ‘Yueyue Fen’ (OB12) and ‘Yueyue Hong’ (OB13). ‘Danhuang Xiangshui Yueji’(O2) was more highly evolved than *R. odorata* var. *odorata* (OO). The upper part of the plot (Lt/St > 1.9) included triploids, tetraploids, and a few of diploids (most with B karyotype) (Fig. 2). Their evolution was more rapid in the direction of the chromosome ratio. The most highly evolved cultivars were ‘Huzhong Yue’ (AC6) and ‘Goldmarie’ (M3). ‘Betty Prior’ (M1), ‘Bao Xiang’ (AC1), and others were more primitive. The karyotype evolution of these four modern rose cultivars did not exceed the range of Ancient Hybrid China roses. The karyotype analysis result is in good agreement with the SSR data.

### FISH with 5 S rDNA probes

**Figure 3:**
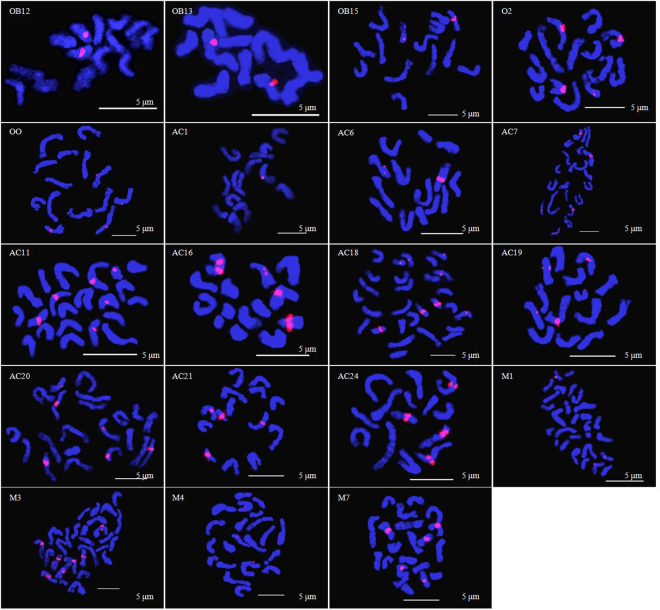
Fluorescence *in situ* hybridization (FISH) analysis using 5 S rDNA (red fluorescence) probes at the metaphase stage of 19 rose cultivars. DAPI (blue).

Using digoxin-labelled 5 S rDNA as a probe, 15 old Chinese garden rose cultivars and 4 modern rose cultivars were used for fluorescence *in situ* hybridization, and the metaphase chromosome karyotype and *in situ* hybridization signals were obtained (Fig. 3). The FISH karyotype ideogram provides a clear visual representation (see Supplementary Fig. S1). 5 S rDNA sites are often located near the centromere of the long arm of the chromosome. There were some differences in the intensity and distribution of signal loci among cultivars.

There were only two 5 S rDNA signals in cultivars of the Old Blush Group and ‘Viridiflora’(AC19), which are all diploid accessions (Fig. 3). Compared to ‘Yueyue Fen’ (OB12) and ‘Yueyue Hong’ (OB13), the distance between 5 S rDNA signals and the centromere was longer in ‘Zhaiye Tengben Yuejihua’ (OB15) and ‘Viridiflora’ (AC19) (see Supplementary Fig. S1). There were still some diploid materials, such as varieties of *R. odorata*, ‘Huzhong Yue’ (AC6), and ‘Si Chun’ (AC18), with four 5 S rDNA signals on two pairs of chromosomes (Fig. 3). The distance between 5 S rDNA signals and the centromere was longer than that of the Old Blush group (see Supplementary Fig. S1). The numbers of 5 S rDNA signals in triploid roses were usually 6 (multiples of three, 3n) or 5 (3n-1); only ‘Betty Prior’ (M2) had 9 (3n). The numbers of 5 S rDNA signals in tetraploid roses were usually 4, 8 (multiples of four, 4n), or 7 (4n-1). Their distances between 5 S rDNA signals and centromeres varied (see Supplementary Fig. S1). The 5 S rDNA signal number increases multiply as the ploidy level increases, but one signal is occasionally lost.

As shown in Supplementary Fig. S1, the 5 S rDNA signal strengths for ‘Yueyue Fen’ (OB12) and ‘Yueyue Hong’ (OB13) were both relatively strong. In other diploid roses, the 5 S rDNA signals were partial and relatively weak. Triploid accessions usually had weak or very weak signals, except for ‘Bao Xiang’ (AC1) and ‘Zixiang Rong’ (AC24), for which a portion of signals was relatively strong. All of the signals were relatively weak or very weak in tetraploids. The 5 S rDNA signal intensity partially and gradually weakened as the ploidy level increased and the karyotype diverged. In sum, the intensity, number and position of 5 S rDNA signals are related to karyotype evolution.

## Discussion

The first goal of this study was to determine whether the Old Blush group is genetically distinct from other ever-blooming China roses. Cultivars of the Old Blush group were clearly separated from other old Chinese garden roses in a dendrogram. Genetic differentiation between the Old Blush group and other old Chinese garden roses was moderate (*F*_ST_ = 0.10). The results show that Old Blush group is distinct from other recurrent blooming old Chinese garden roses. Cultivars in Old Blush group were closely related to each other not only at the phenotypic level, but also at the molecular level. The Old Blush group should have an independent classification status among China roses. Soules named eight accessions (e.g. ‘Climbing Old Blush’, ‘Viridiflora’ and ‘Single Pink’) in the Old Blush group based on their SSR profiles, which were identical to that of ‘Old Blush’^10^. Climbing-type and single flower-type Old Blush roses were also examined in this study, but only ‘Viridiflora’ (AC19) had an identical SSR profile to that of ‘Old Blush’ (OB12). This difference may be explained by the different accessions and SSR makers. As the type specimen, ‘Yueyue Fen’ was named *R. chinensis* var. *chinensis*, and is a cultivar, not a wild specimen^5^. ‘Yueyue Fen’ has diverse horticultural cultivars, and these are the most common ancient roses with the longest history (from the Song dynasty, 960–1279 DC) of cultivation in China^6,35^. The Old Blush group is generally considered the oldest and most common type of ever-blooming ancient China rose.

*R. chinensis* var. *spontanea* is commonly considered one of the wild ancestors of ‘Old Blush’ based on morphological characters^35,36^ and chloroplast sequence haplotypes^10,12^. *R. chinensis* var. *spontanea* (CS1, CS2) clustered with sect. Synstylae and most of the species were separated from rose cultivars (Fig. 1), confirming previous results obtained by Soules^10^ and Qiu *et al.* ^37^. According to Qiu *et al*., wild roses are separated from old garden roses and modern roses^37,38^. Compared to other accessions, *R. lucidissima* (L.) is relatively genetically more closely related to the Old Blush group (Fig. 1). This result supports the hypothesis that *R. lucidissima* is involved in the breeding process for *R. chinensis* var. *chinensis* ‘Old Blush’^35^. The chloroplast data showed that *R. chinensis* var. *spontanea* is a maternal ancestor of the China Roses^10^. As a maternal ancestor, the genetic material in the nucleus of *R. chinensis* var. *spontanea* may be diluted by multiple hybridization events. This may explain why *R. chinensis* var. *spontanea* was not closely related to the Old Blush group based on SSR data.

The difference in karyotypes among cultivars within the same species seems to arise from divergence between parental accessions and offspring in phyletic evolution^39^. ‘Viridiflora’ had been speculated to be a sport of ‘Old Blush;’ they were genetically identical in this study and in several previous studies^7,10,15^. However, ‘Viridiflora’ (AC19) was more highly evolved than ‘Yueyue Fen’ (OB12) (Fig. 2), implying that ‘Viridiflora’ is a sport of ‘Old Blush; the latter has 12 m (metacentric chromosomes) and 2 sm (submedian metacentric chromosomes), while ‘Viridiflora’ has 10 m and 4 sm (Table 2). If ‘Viridiflora’ is indeed a sport of ‘Old Blush’, structural variation occurred in two chromosomes. ‘Zhaiye Tengben Yuejihua’ (double flower, once flowering climbing shrub) was presumed to be an intermediate between the wild ancestor *R. chinensis* var. *spontanea* and ‘Old Blush’^12^. A karyotype analysis showed that ‘Zhaiye Tengben Yuejihua’ (OB15) is more highly evolved than ‘Yueyue Hong’ and ‘Yueyue Fen’ (Fig. 2). This result suggests that ‘Zhaiye Tengben Yuejihua’ is derived from ‘Old Blush’.

The second goal of this study was to determine whether the Ancient Hybrid China group was bred on the basis of Old Blush group, Odorata group and species. SSR data revealed that roses of the Ancient Hybrid China group were located midway between the Old Blush group, Odorata group and species, probably as a consequence of their hybridization history (Fig. 1). From the perspective of ploidy levels, triploid roses in the Ancient Hybrid China group appear to be the result of hybridization between diploid roses (Old Blush group and Odorata group) and tetraploid roses (some species roses). Previous studies have also reported that triploid roses are midway between diploids roses and tetraploids roses based on principal component analyses, possibly as a consequence of their hybridization^5^. There are also diploids and tetraploids in the Ancient Hybrid China group, as well as tetraploid modern roses, which are probably the products of different ploidy combinations. Unreduced gametes produced by triploids may have formed tetraploid roses^40^. From the perspective of genetic differentiation, the genetic distances to species roses become smaller from the Old Blush group to the Odorata group, then to the Ancient Hybrid China group and modern roses. Accordingly, the Ancient Hybrid China group and modern roses might be the result of the continuous hybridization of the Old Blush group, Odorata group and wild species. The genetic background of modern roses is similar to that of the Ancient hybrid China group, as evidenced by the low differentiation (*F*_ST_ = 0.01).

From the perspective of karyotype evolution, the karyotype of Old Blush roses and *R. odorata* (mainly 1 A and 2 A) were more primitive than those of Ancient Hybrid China roses and modern roses (mainly 1B and 2B). Plants are more evolved when their karyotype asymmetry is higher^41–43^. A scatter diagram of rose cultivars based on average arm ratio and length ratio (Lt/St) suggest that polyploid Ancient Hybrid China roses and modern roses evolved more rapidly along the direction of chromosome length ratio. The Old Blush group and Odorata group are more primitive than the Ancient Hybrid China group and modern roses along the direction of chromosome length ratio. Differences in chromosome length ratio are caused by variation in chromosome structure (i.e., deletion, duplication and translocation events). Therefore, changes in chromosome structure may explain the formation of Ancient Hybrid China roses and modern roses. The hybridization between diploid roses (Old Blush group and Odorata group) and tetraploid roses (some species) may promote variation in chromosome structure. These results were consistent with those of Jian *et al*.^31^, who showed that compared to *R. chinensis* var. *chinensis* and *R. odorata*, other old Chinese garden roses and modern roses have more variation in chromosome structure and number. *R. odorata* var. *odorata* (OO) and ‘Danhuang Xiangshui Yueji’ (O2) are more highly evolved than ‘Yueyue Fen’ (OB12) and ‘Yueyue Hong’ (OB13) with respect to both chromosome length ratio and arm ratio, indicating that the Odorata group is more highly evolved than the Old Blush group. ‘Yueyue Fen’ (OB12) is the most primitive cultivar among all accessions (Fig. 2). The evolutionary relationships among groups confirmed that the Ancient Hybrid China group may have been bred on the basis of the Old Blush group and Odorata group.

The third aim of this study was to determine whether the physical localization of 5 S rDNA genes was related to the genetic relationships and karyotype evolution of rose cultivars. The location and number of rDNA loci on chromosomes can effectively reflect the degree of differentiation among species^44–48^. When the genetic relationship between plant materials is closer, the evolutionary distance is smaller and the distribution of rDNA is more similar^49^. Moreover, unlike 45 S rDNA, which is closely associated with nucleolar organizer regions, the location of the 5 S gene in rDNA regions is more diverse^50^. In the process of polyploidization, for each additional set of chromosomes, one rDNA locus is added, where one set of chromosomes corresponds to one rDNA locus^22^. The number of 5 S rDNA signals in this study showed that one set of chromosomes corresponded to one, two or three rDNA loci, and sometimes one signal was lost. Mishima *et al*. also found that diploid rose (*R. multiflora*) had four 5 S rDNA sites^51^. Chromosome rearrangements, including duplication and translocation, may lead to 5 S rDNA signal increases in multiples. The loss of one signal could be due to the loss of a chromosome fragment by unequal crossing over^52^ and transposition^53^ in the process of polyploidization. In addition, Fernández-Romero *et al*. suggested that it may be due to the allopolyploid nature of accessions^32^. Because a higher ploidy level indicates a more evolved karyotype (Table 2), the increase of 5 S rDNA sites implies more highly evolved rose cultivars.

Based on a karyotype analysis and fluorescence *in situ* hybridization, when an accession (Old blush group and Odorata group) had a more primitive karyotype, it had fewer 5 S rDNA signals, stronger signal intensities and signals closer to the centromere. During the evolution of the karyotype (the Ancient Hybrid China group and modern roses), 5 S rDNA signals increased in number, decreased in intensity, and exhibited variation in the distance from the centromere. These patterns are consistent with those reported by Jian *et al*.^31^, who found that compared to *R. chinensis* var. *chinensis* and *R. odorata*, other old Chinese garden roses and modern roses are more diverse with respect to the number and intensity of 4 5 S rDNA signals. FISH is a semi-quantitative technique; and the actual number of gene copies cannot be directly determined, but the size and strength of the hybrid signal indirectly reflects the number of gene copies^54^. The 5 S rDNA signal intensity decreased during evolution, and this may be due to the decrease in 5 S rDNA copy number during the process of rose crossbreeding. Chromosome variation, including chromosome inversions and translocations, may lead to 5 S rDNA sites that are further away from the centromere.

In analyses of plant evolution, it may be more effective to combine molecular marker techniques and fluorescence *in situ* hybridization. Han *et al*.^55^ used FISH and random amplified polymorphic DNA (RAPD) to analyse the evolution of *Vicia ramuliflora* at the diploid and tetraploid stages. In this study, the number, intensity and distribution of 5 S rDNA signals were related to the karyotype evolution of rose groups, which were classified according to SSR data. The Old Blush group is the most primitive rose group; roses in this group had the fewest 5 S rDNA sites, the strongest signal intensity and the smallest distances from 5 s rDNA sites to the centromere. The Odorata group is more highly evolved than the Old Blush group with respect to both chromosome length ratio and arm ratio; roses in this group had more 5 S rDNA sites, weaker signal intensities and greater distances from the centromere. The Ancient Hybrid China group and modern roses evolved from the Old Blush group and Odorata group, mainly in the direction of chromosome length ratio. They had the most 5 S rDNA sites, the weakest signal intensity and uneven distances from the centromere.

## Methods

### Plant materials and DNA extraction

In total, 81 accessions were chosen, including 46 old Chinese garden roses. For comparison, we also selected 7 modern rose cultivars, 1 *R. rugosa* cultivar and 27 wild roses from 7 sections in subgenera *Rosa* (see Supplementary Table S1). The healthy fresh young leaves of each accession were harvested, deep frozen in liquid nitrogen and stored at −80 °C in a freezer. Total genomic DNA was extracted using the Fast DNA Kit (Tiangen Biotech, Beijing, China) according to the manufacturer’s instructions. The ploidy of 19 accessions (Table 2) was obtained by fluorescence *in situ* hybridization, and young, fresh leaves of other accessions (see Supplementary Table S1) were measured by flow cytometry. ‘Yueyue Fen’ (for which ploidy was obtained by fluorescence *in situ* hybridization) was used as an internal reference standard. A 1-cm^2^ piece of each material was chopped up in 500 μL of cell lysis buffer in a Petri dish. Then, 1.5 mL of cell lysis buffer mixed with DAPI was added. A 30-μm nylon mesh was used to filter the suspension. A Partec PA II flow cytometer (Partec GmbH, Münster, Germany), equipped with a mercury arc lamp (HBO/100), was used to analyse fluorescence.

### Microsatellite marker genotyping

Twenty-two markers (see Supplementary Table S3) were selected from 42 published genome-wide SSR markers^45^ by polymorphism analyses in a subset of ten accessions. The forward primers for the 22 markers were labelled with FAM, HEX, TAMRA, and ROX fluorescent dyes. Twenty-two SSR markers amplified successfully across all 81 accessions and showed high polymorphism by capillary electrophoresis. Genotyping was executed on an ABI 3730 DNA analyser (Applied Biosystems, Foster City, CA, USA). Amplification reactions for ABI were performed in 10 μL containing 8 ng of DNA, 5 μL of multiplex master mix kit (QIAGEN, Hilden, Germany), 4 pmol each forward (labelled) and reverse primer. The PCR procedure was as follows: 5 min at 95 °C, followed by 35 cycles of 30 s at 94 °C, 30 s at 56 °C and 30 s at 72 °C and 10 min at 72 °C for a final extension. Then, 1 μL of 100× diluted PCR product was mixed with the GeneScan-500 LIZ size standard (Applied Biosystems) and Hi-Di formamide (Applied Biosystems) and then run on an ABI 3730DNA analyser. Genemapper 4.0 (Applied Biosystems) was used to analyse the output from the ABI platform.

### Genetic diversity and distance

The “allelic phenotype” was determined for each locus using data recorded in a binary data matrix (1 for present and 0 for absent)^8,56,57^. To assess diversity, the number of observed alleles (*A*_*o*_), the mean number of alleles per individual (*A*_*m*_)^6^ and the effective number of alleles (*A*_*e*_)^58^ were calculated for each SSR locus. NTSYS version 2.10 was used to implement unweighted pair-group method with arithmetic means (UPGMA) clustering in order to assess and visualize genetic relationships among genotypes. We used the fixation index (*F*_ST_) and expected heterozygosity (*H*_*e*_) to estimate diversity. Genetic distance was measured by *F*_ST_ based on allele frequency differences among accessions^59^. *H*_*e*_ refers to the probability that two randomly chosen alleles at a specific locus within a set of genotypes will be different under Hardy–Weinberg equilibrium (i.e., assuming random mating). The genetic differentiation (*F*_ST_) and expected heterozygosity (*H*_*e*_) were calculated using SPAGeDi 1.3, which analyses ploidy level data^60^.

### Chromosome preparation

Nineteen typical cultivars were chosen from the 81 accessions to represent each horticultural group of old Chinese garden roses and modern roses. There were 3 cultivars from Old Blush group, 2 cultivars from the Odorata group, 10 cultivars from the Ancient Hybrid China group and 4 cultivars from modern roses (Table 4). Somatic metaphase chromosome spreads were prepared from fresh shoot apical meristems of 19 accessions and pretreated according to the methods of Ding *et al*.^30^. Briefly, fresh young shoot apical meristems were treated with 0.002 M 8-oxyquinoline for 4 h in dark conditions at room temperature, then fixed in freshly prepared ethanol: acetic acid (3: 1, v/v) for 24 h. The shoot apical meristem was isolated, washed 3–5 times with distilled water and enzymatically digested in 2% pectinase plus 4% cellulose at 37 °C for 2–3 h. Subsequently, the samples were squashed with 1–2 drops of 45% acetic acid after 30 min of immersion in distilled water. The samples were frozen in liquid nitrogen for 5 min, and the slides were air dried and stored at −20 °C.

### Probe DNA preparation

5 S rDNA was amplified from total genomic DNA of *R. multiflora*. Amplification was carried out with a pair of 5 S rDNA-specific primers: forward (5′ → 3′)GAGAGTAGTACTAGGATGGGTGACC, reverse (5′ → 3′) CTCTCGCCCAAGAACGCTTAACTGC. The 25-μL reaction mix contained 0.6 μL of template DNA, 12.5 μL of 2 × Taq PCR Master Mix and 0.5 μL of forward and reverse primers. Amplification cycles were performed as follows: 94 °C, 5 min; (94 °C, 30 s; 53.5 °C, 30 s; 72 °C, 1 min) × 30; 72 °C, 5 min.

### Fluorescence ***in situ*** hybridization analysis

Fluorescence *in situ* hybridization was performed according to the methods described by Ding *et al*.^30^. Briefly, the slides were pretreated with RNase A for 1 h in a constant temperature humidity chamber at 37 °C, washed in 2 × SSC for 10 min, fixed in 4% paraformaldehyde solution for 10 min and then dehydrated in a −20 °C pre-cooled ethanol series. The hybridization mixture, containing 20 × SSC (Saline Sodium Citrate), 50% deionized formamide (v/v), 50% dextran sulphate (w/v) and 3 μL of 5 S rDNA probe, was denatured at 80 °C for 10 min and then incubated at 37 °C overnight (14–18 h) in constant temperature humidity chamber. After hybridization, the slides were washed in 2 × SSC at 42 °C for 10 min and then digoxygenin-labelled probes were detected using FITC-conjugated anti-digoxygenin antibodies (Roche, Mannheim, Germany).

### Chromosome observation

The chromosomes were counterstained with DAPI using Vectashield (Vector Laboratories, Inc., Burlingame, CA, USA) and evaluated under the Olympus BX-51 fluorescence microscope. Images were captured using Cytovision and then processed with Photoshop software (Adobe Systems Software Ireland Ltd., version 13.0.0.0). The nomenclature for chromosome morphology, arm ratio, karyotype and karyotype formula are described by Levan^61^ and Stebbins^42^. The asymmetrical karyotype index was calculated following the methods of Arano^41^. The relative length of chromosomes was determined by the method of Kuo *et al*.^62^. An increase in karyotype asymmetry can reflect unequal lengths of chromosome arms or sizes of different chromosomes in the same nucleus^43^. The former is referred to as the average arm ratio, and the latter is referred to as the length ratio (Lt/St). With the average arm ratio as the abscissa and the chromosome length ratio as the ordinate, plant materials in the Cartesian coordinate system were plotted to evaluate their relative evolutionary positions^63^. Consequently, the evolution of plant karyotypes showed a bidirectional trend (chromosome length ratio and arm ratio)^44^. The average arm ratio was plotted against the chromosome length ratio using Excel (Fig. 2).

### Data availability statement

All data generated or analysed during this study are included in this published article (and its Supplementary Information files).

## Electronic supplementary material

Supplementary Information
