# Supplementary material for: Genetic relationships and evolution of old Chinese garden roses based on SSRs and chromosome diversity
Source: Sci Rep. 2017 Nov 13;7:15437. doi: 10.1038/s41598-017-15815-6 (PMC5684293; doi:10.1038/s41598-017-15815-6)
Supplement: Supplementary file 1 — Supplementary Information [file 41598_2017_15815_MOESM1_ESM.pdf]

Genetic relationships and evolution of old Chinese garden roses based on SSRs and chromosome diversity

Jiongrui Tan[1], Jing Wang[1], Le Luo[1], Chao Yu[1], Tingliang Xu[1], Yuying Wu[1], Tangren Cheng[1], Jia Wang[1], Huitang Pan[1]*, and Qixiang Zhang[1]

Supplementary Figure S1. FISH karyotype ideogram summarizing the 5SrDNA signals (red fluorescence) of 19 Rose culitivars.

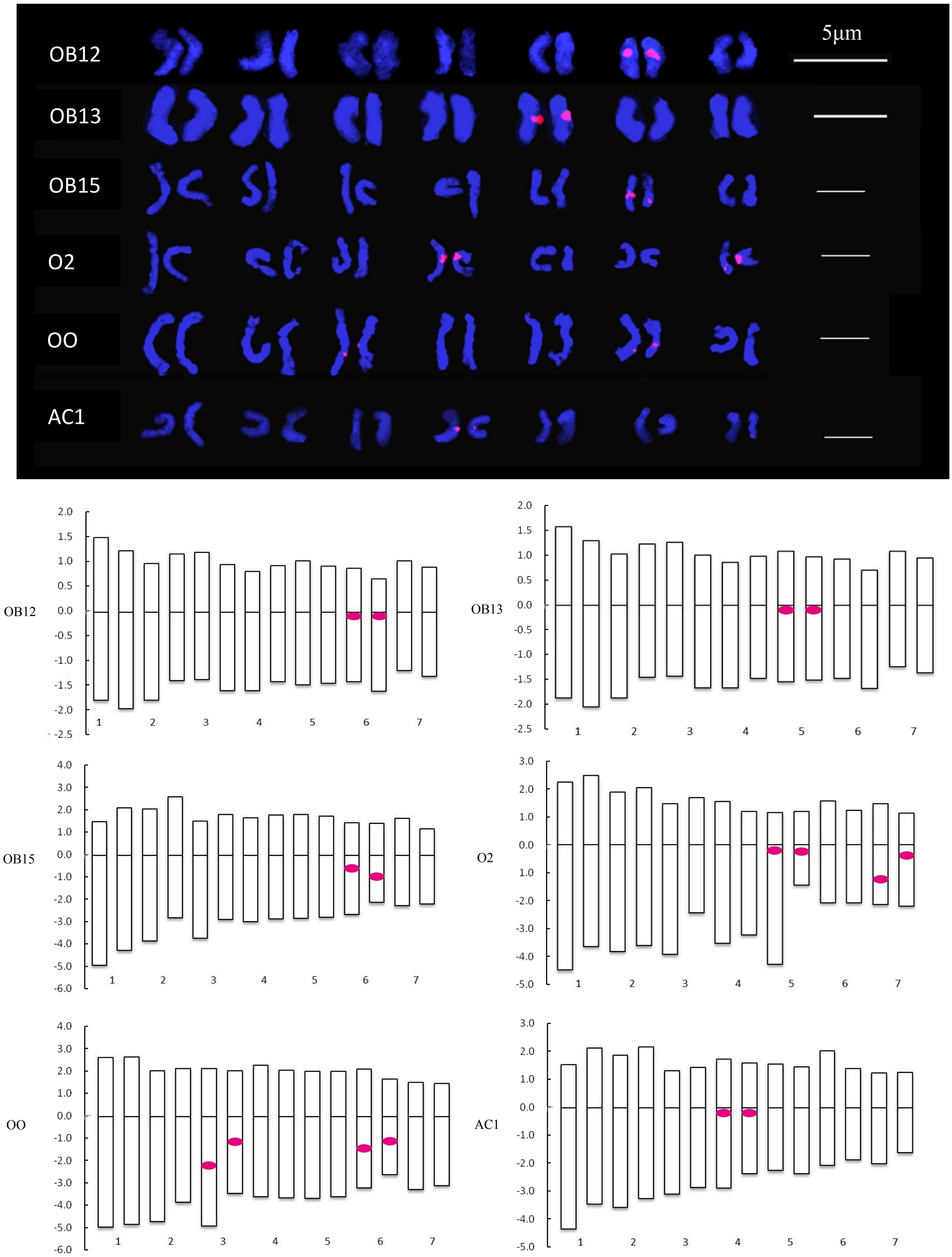

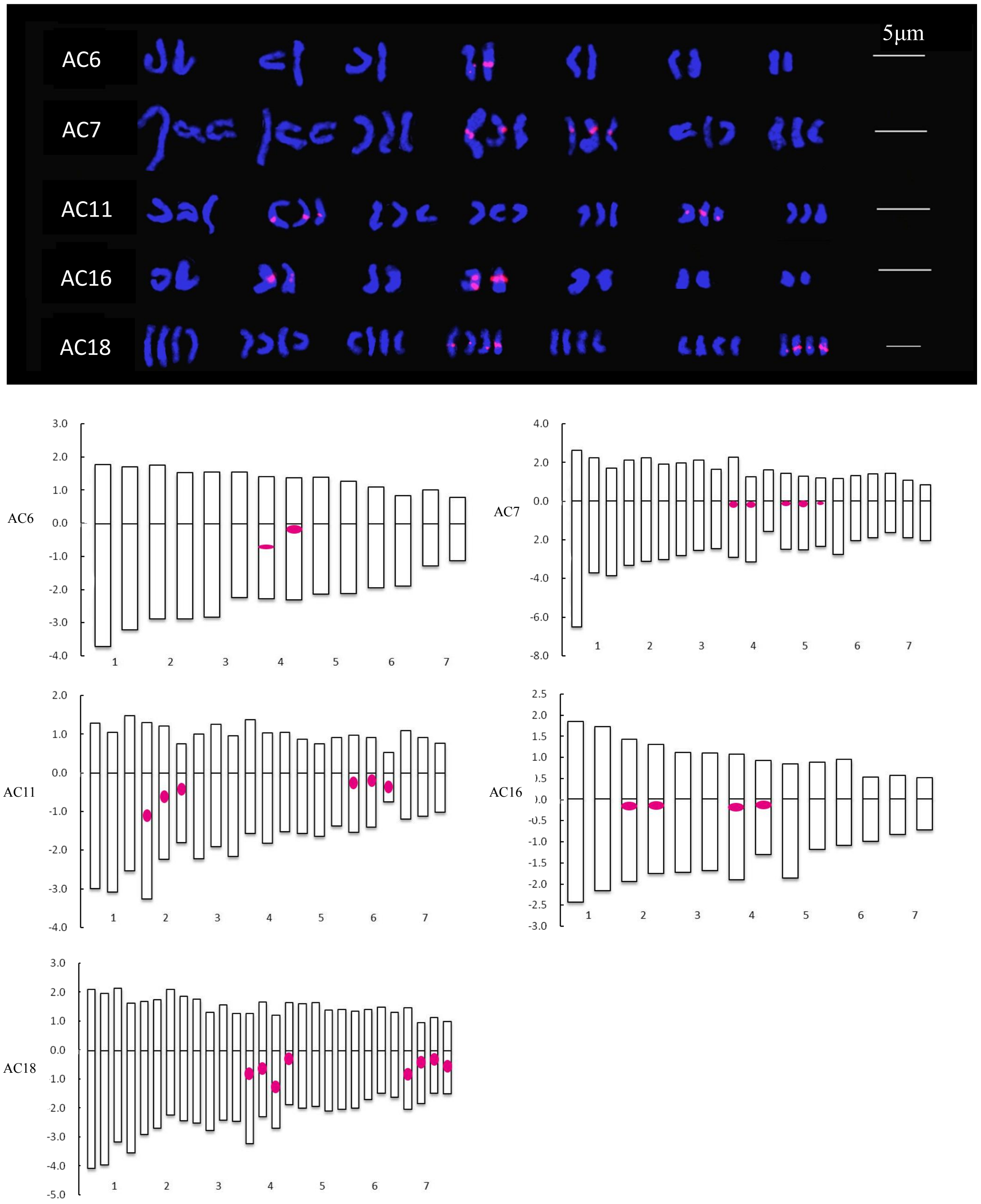
AC6
AC7
AC11
AC16
AC18
5μm

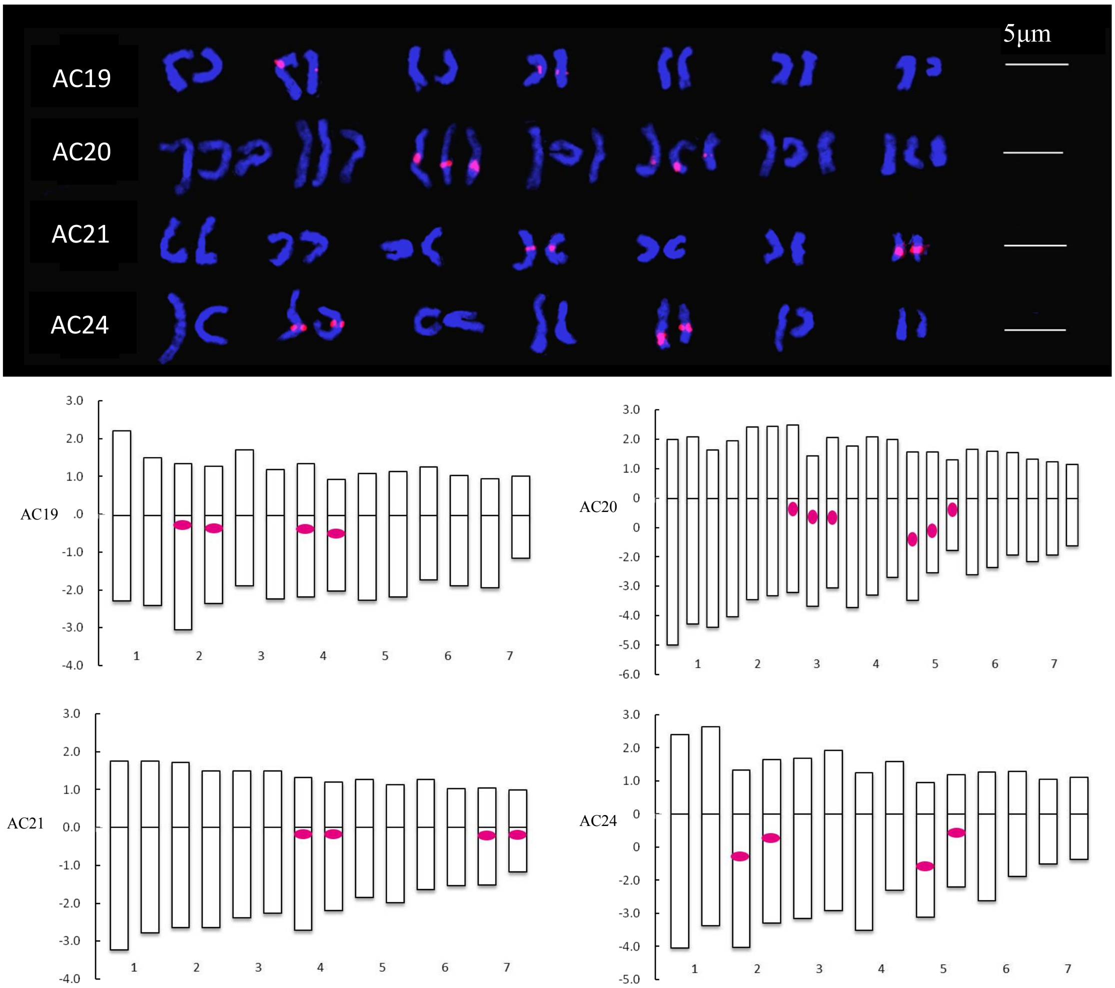
AC19
AC20
AC21
AC24
5μm

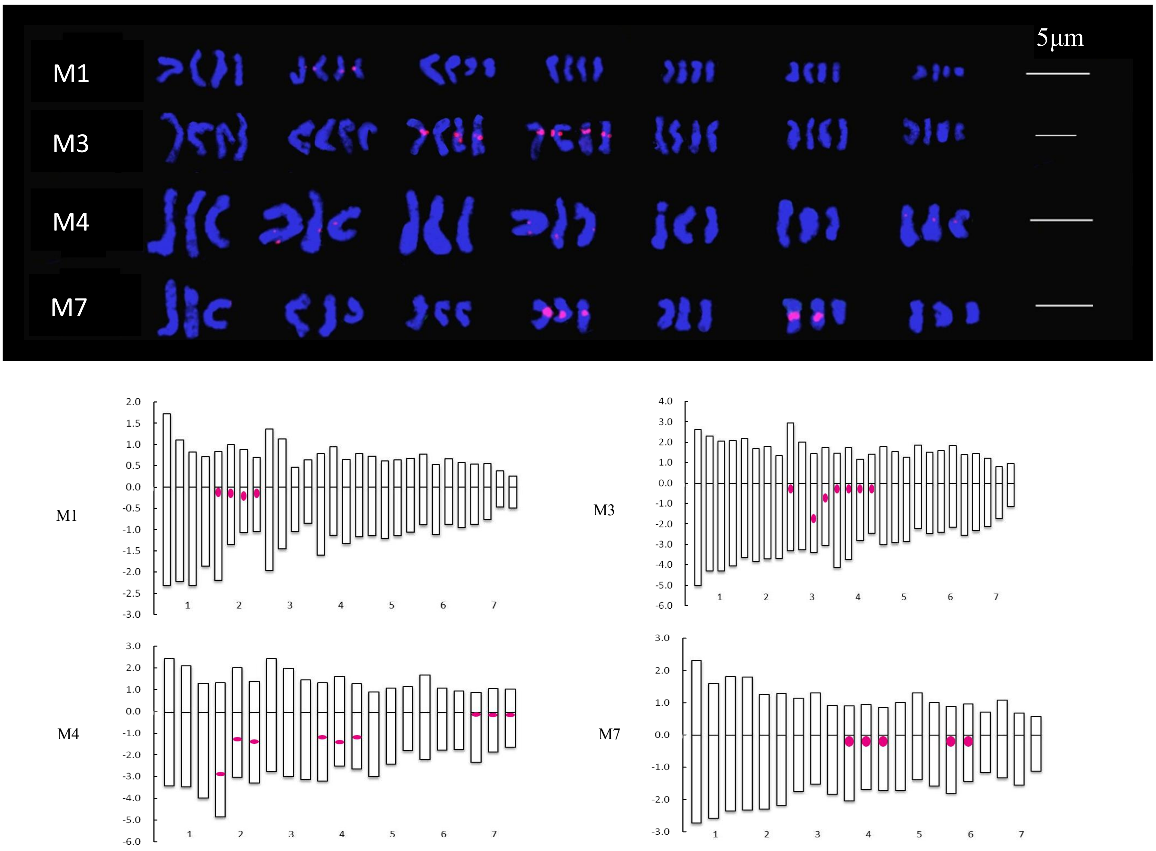
M1
M3
M4
M7
5μm

Supplementary Table S1. Information of 81 Rose accessions. [x] Names of accessions are listed as given by the source, and the grouping principle of Old Blush group and Odorata group refer to *Old Roses in China*[14]. [Y] KYC: Kunming Yang Chinese Rose Gardening Co., Ltd. Kunming, Yunnan, China. NERCF: Nursery of National Engineering Research Center for Floriculture, Beijing, China.

**Cultivar or species name**[x]	**Sample number**	**Ploidy**	**Source**[y]
**Old Blush Group, Sect. Chinenses, *Rosa chinensis* var. *chinensis***			
‘Bianse Yueyue Hong’	OB1	2	KYC
‘Dahua Tengben Yueyue Hong’	OB2	2	KYC
‘Jianlei DahuaYueyue Hong’	OB3	2	KYC
‘Luguhu Yuejihua’	OB4	2	KYC
‘Teng Yueyue Hong’	OB5	2	KYC
‘Tengben Danban Yueyue Hong’	OB6	2	KYC
‘Tieban Yueyue Hong’	OB7	2	KYC
‘Tuanlei DahuaYueyue Hong’	OB8	2	KYC
‘Wuyuan Yueyue Hong’	OB9	2	KYC
‘Xiaohua Tengben Yueyue Fen’	OB10	2	KYC
‘Yueyue Fen’1 (Old Blush)	OB11	2	KYC
‘Yueyue Fen’ 2 (Old Blush)	OB12	2	NERCF
‘Yueyue Hong’ 1 (Slater’s Crimson China)	OB13	2	NERCF
‘Yueyue Hong’ 2 (Slater’s Crimson China)	OB14	2	KYC
‘Zhaiye Tengben Yuejihua’	OB15	2	NERCF
‘Zhongguo Fen’	OB16	2	KYC
**Ancient Hybrid Chinas group, Section Chinenses, *R. chinensis***			KYC
‘Bao Xiang’	AC1	3	KYC
‘Da Fugui’	AC2	3	KYC
‘Fenxiang Lou’	AC3	3	KYC
‘Honglian Yuejihua ’	AC4	2	KYC
‘Huang Furong ’	AC5	4	KYC
‘Huzhong Yue ’	AC6	2	KYC
‘Jinfen Lian’	AC7	4	KYC
‘Jinou Fanlv ’	AC8	3	KYC
‘Ju Nang’	AC9	2	NERCF
‘Liyun Taohua’	AC10	3	KYC
‘Mutabilis1’	AC11	2	KYC
‘Mutabilis2’	AC12	2	NERCF
‘Nanuo Yueji Hua’	AC13	2	KYC
‘Qinglian Xueshi’	AC14	3	KYC
‘Ruan Xianghong ’	AC15	4	NERCF
‘Sai Zhaojun’	AC16	2	KYC
‘Shui Meiren ’	AC17	3	KYC
‘Si Chun’	AC18	2	KYC
‘ViridiflM4ora’	AC19	2	KYC
‘Yingri Hehua’	AC20	3	KYC
‘Yu Linglong’	AC21	2	KYC
‘Yunzhen Xiawei’	AC22	4	NERCF
Yushi Zhuang’	AC23	3	KYC

'Zixiang Rong'	AC24	3	KYC
**Odorata group, Sect. Chinenses, *R. odorata***			
'Dahua Fenhong Xiangshui Yueji'	O1	2	KYC
'Danhuang Xiangshui Yueji '	O2	2	KYC
'Siji Danhuang Xiangshui Yueji '<br>('Parks' Yellow Tea-Scented China')	O3	4	KYC
'Siji Fenhong Xiangshui Yueji'	O4	2	KYC
'Ziyun Xiangshui Yueji'	O5	2	KYC
**Modern roses, Sect. Chinenses**			
'Betty Prior'	M1	3	KYC
'Double Delight'	M2	4	KYC
'Goldmarie'	M3	4	KYC
'Honglian Wu'	M4	3	NERCF
'Pink Peace'	M5	4	KYC
'Porcelina'	M6	4	NERCF
'Princesse de Monaco'	M7	4	KYC
**Species roses in Sect. Chinenses**			
R. chinensis var. spontanea 1	CS1	2	KYC
R. chinensis var. spontanea 2	CS2	2	NERCF
R. lucidissima	L	2	KYC
R. odorata var. gigantea	OG	2	KYC
R. odorata var. odorata	OO	2	KYC
**Sect. Synstylae**			
R. glomerata	S1	2	KYC
R. lichiangensis	S2	2	KYC
R. longicuspis	S3	2	KYC
R. multiflora var. albo-plena	S4	2	NERCF
R. multiflora var. carnea	S5	2	KYC
R. multiflora var. multiflora	S6	2	KYC
R. soulieana	S7	2	KYC
**Sect. Microphyllae**			
R. roxburghii	MP	2	NERCF
**Sect. Pimpinellifoliae**			
R. platyacantha	P1	2	KYC
R. primula	P2	2	KYC
R. spinosissima	P3	2	KYC
R. xanthina	P4	2	NERCF
R. xanthina var. normalis	P5	2	NERCF
**Sect. Banksianae**			
R. banksiae	B1	2	KYC
R. banksiae var. normalis	B2	2	KYC
R. cymosa	B3	2	KYC
**Sect. Laevigatae**			
R. laevigata var. laevigata	L1	2	KYC
R. laevigata var. semiplena	L2	2	KYC
**Sect.Cinnamomeae**			
R. laxa 7	C1	4	KYC

R. laxa 34	C2	4	KYC
R. macrophylla var. glandulifera	C3	4	KYC
R. oxyacantha	C4	4	KYC
R. rugosa	C5	2	NERCF
**Cultivar of rugosa**			
'Dahong Zizhi'	RC	2	NERCF

Supplementary Table S2. Indexes of genetic diversity observed in 81 accessions studied with 22 SSRs. LG, linkage group; No. observed, number of observed genotypes; A_o, number of observed alleles; A_e, effective number of alleles; A_m, mean number of alleles per individuals; H_e, expected heterozygosity.

SSR	LG	Motif	No. observed	A_o	A_m	A_e	H_e
327	Y4	$(CTT)_6$	81	8	1.7	1.7	0.798
353	LG5	$(TC)_7$	77	6	1.6	1.6	0.715
387	LG2	$(CT)_9$	81	15	2.1	1.3	0.823
397	LG4	$(CT)_{14}$	80	18	2.0	1.9	0.865
405	LG7	$(ATG)_5$	81	16	1.8	1.4	0.788
464	Y12	$(TCGGA)_3$	81	5	1.3	1.0	0.337
490	LG6	$(TCT)_6$	80	12	1.8	1.3	0.731
509	LG7	$(CAC)_5$	80	13	1.3	1.2	0.418
510	LG2	$(AAG)_5$	81	4	1.3	1.0	0.281
541	LG6	$(AG)_7$	78	11	1.8	1.9	0.823
596	LG5	$(AGG)_5$	78	6	1.6	1.2	0.677
625	LG7	$(TC)_8$	75	13	2.0	1.9	0.858
637	LG7	$(TTGATT)_3$	77	5	1.8	1.2	0.735
648	LG6	$(CT)_8$	80	12	2.0	1.4	0.818
651	LG6	$(CAG)_5$	80	6	1.7	1.2	0.698
682	LG3	$(TC)_{10}$	80	12	1.9	1.3	0.765
695	LG3	$(TA)_8$	79	10	1.9	1.4	0.803
CL2996	LG2	$(CCG)_{17}$	81	9	1.9	1.3	0.785
H22E04	LG6	$(AAG)_7$	80	5	1.9	1.0	0.689
Rw5G14	LG7	$(CT)_7(C)_8$	80	11	1.9	1.8	0.859
Rw10M24	LG7	$(CT)_7(TA)_4$	80	19	1.8	1.9	0.829
Rw22A3	LG6	$(TTC)_6$	81	11	2.2	0.9	0.757
Totle			81	227		30.9	
Mean				10.3	1.8	1.4	0.721

Supplementary Table S3. Information of 22 SSR primer pairs used in this study.

SSR name	Annealing temperature	Expected size （bp）	Forward/Reverse（5'→3'）
327	59.8	277,232, 202	F: ACTCCTCCAAAGCTTCACCA<br>R: CCTCATCGACAGAGTCGTCA
353	59.7	212,107,223	F: CGCCCTAGTCTGCTCTCTCTC<br>R: CTCAAGCTGAAGCTCGGAGT
387	58.7	202,229,223	F: GCACTCTTGACGTTGTCCAT<br>R: GTCAATGTAGTCCGGTTCGG
397	59.9	222,221,252	F: GGCCTAGCAAAGCAACAAAC<br>R: AGTGG AGGGCAGTCTCTGAA
405	59.9	265,183,237	F: CAGCGAAAAGAACAAGGACC<br>R:CAGAAGCTAATAAATTAACAATCACCA
464	59.5	134,250,149	F: TCTTTCGGTTCAGAAAGTTCG

			R: CTCGCTGATCTTGTCCATCA
490	60.0	128,125,272	F: ACAACCAACCCAAGAACTCG<br>R: TGCCAGCTTCAGTCTCACCT
509	60.0	208,252,261	F: CAACTGGGTTGGGTCAGTCT<br>R: TCAAATGTACCTTGCGCTTG
510	60	190,157,156	F: AGAGGTTTAGGGCAGCCATT<br>R: GCGAATGATGGTGGAGAGTT
541	59.7	241 ,243,242	F: CTACTCCAATGTCCGCTTCC<br>R: GTTGGAGAAGAAGCCGTGAG
596	60.6	211,212, 222	F: CGAGGAAAAACCCAAAATCC<br>R: TGGAAGCAAGAAAAGGCAGT
625	59.0	143,213,142	F: GGCGTCTCTCACATCTCAAA<br>R: AAGATCTTCTCTCCGGCCTT
637	60	280,279,280	F: GCCGTAATTCGTGGAAAGAA<br>R: ATGCCACCAGAACCTTGAAC
648	60.5	167,169,217	F: CCTAAAGCTTAAGCCCCCAA<br>R: GCAATAGACTTGGCAGCCTC
651	60.2	166,167,165	F: TCTGAGCACGACTCAACAGG<br>R: AGGCATGTAATGCTGTGGGT
682	59.7	205,131,207	F: TTCTTGAGCTAAAAGTGCATCG<br>R: CAGATCCAAACCGAACCCTA
695	59.8	248,244,264	F: AGAAAAGCGAAAGCACAAGC<br>R: CTTAAATGCGCCACCAATTT
CL2996	55.0	183	F: GCCACCATAGCCAGAGACAT<br>R:GGGCAGAGAAGAAGTTGACG
H22E04	55.0	241	F: GACATCACCACCACCACAAG<br>R: AACCAAGGTTTCCAGTTCCA
Rw5G14	55.0	231	F: TGGTTTGGGGTTTTGTGTCT<br>R: GCACAGTCTCCACCTGACAA
Rw10M24	53.5	252	F: TTAATCCAAGGTCAAAGCTG<br>R: TCTCTTTCCCTCCTCACTCT
Rw22A3	52.9	150	F: AGAGAATTGAAAAGGGCAAG<br>R: GAGCAAGCAAGACACTGTAA
